# Diversity and Antimicrobial Properties of Lactic Acid Bacteria Isolated from Rhizosphere of Olive Trees and Desert Truffles of Tunisia

**DOI:** 10.1155/2013/405708

**Published:** 2013-09-14

**Authors:** Imene Fhoula, Afef Najjari, Yousra Turki, Sana Jaballah, Abdelatif Boudabous, Hadda Ouzari

**Affiliations:** Université de Tunis El Manar, Faculté des Science de Tunis, LR03ES03 Laboratoire Microorganismes et Biomolécules Actives, 2092 Tunis, Tunisia

## Abstract

A total of 119 lactic acid bacteria (LAB) were isolated, by culture-dependant method, from rhizosphere samples of olive trees and desert truffles and evaluated for different biotechnological properties. Using the variability of the intergenic spacer 16S-23S and 16S rRNA gene sequences, the isolates were identified as the genera *Lactococcus, Pediococcus, Lactobacillus, Weissella,* and *Enterococcus*. All the strains showed proteolytic activity with variable rates 42% were EPS producers, while only 10% showed the ability to grow in 9% NaCl. In addition, a low rate of antibiotic resistance was detected among rhizospheric enterococci. Furthermore, a strong antibacterial activity against plant and/or pathogenic bacteria of *Stenotrophomonas maltophilia, Pantoea agglomerans, Pseudomonas savastanoi*, the food-borne *Staphylococcus aureus,* and *Listeria monocytogenes* was recorded. Antifungal activity evaluation showed that *Botrytis cinerea* was the most inhibited fungus followed by *Penicillium expansum, Verticillium dahliae,* and *Aspergillus niger*. Most of the active strains belonged to the genera *Enterococcus* and *Weissella*. This study led to suggest that environmental-derived LAB strains could be selected for technological application to control pathogenic bacteria and to protect food safety from postharvest deleterious microbiota.

## 1. Introduction

Given the world's growing demand for food, more attention is needed for food preservation, postharvest, and agricultural product preservation from different harmful factors such as the contamination caused by microbial spoilage and toxic metabolites produced by yeast, mold, and/or bacteria [1, 2], as well as the extensive use of synthetic chemicals and pesticides in food and agriculture. These factors may pose a health risk for human and animals and affect the ecological equilibrium of the environment [3].

Therefore, there is growing interest to establish alternative bioproducts to replace chemicals and toxic pesticides. For this purpose, using bacteria or natural compounds which exhibit the same inhibitory effect on phytopathogenic and spoilage microbes was not only shown to be efficient in storage life extension and nutritive and safety value retention, of food products but also the environment safeguarding [4, 5]. Such bacteria are known by “biological control agents” [6]. 

Lactic acid bacteria form an ecologically heterogeneous group of Gram-positive bacteria, nonspore forming, immobile, and catalase negative, that excretes lactic acid as major end product and generally recognized as safe (GRAS) organisms [7]. They are also selected as probiotic, which are able to promote health and prevent infections against enteropathogenic bacteria [8, 9]. LAB are usually harbor carbohydrate-rich environments and found in various food products such as milk, plant, meat, intestinal mucosa of human, and animals [8, 10] but especially proliferate in different fermented foods [11]. Owing to particular physiological and biochemical traits, such as exopolysaccharide production, organic acids, aromatic compounds, tolerance to low water activity, and antimicrobial production [5, 12, 13], LAB found different industrial applications, either by their biopreservatives or techno-functional properties [14]. In fact, many authors reported that some LAB strains are able to inhibit food-borne pathogens such as *Staphylococcus aureus, Salmonella typhimurium, Escherichia, coli* and *Listeria monocytogenes* [15, 16]. In addition, LAB are efficient to inhibit mycotoxicogenic fungi (*Penicillium expansum, Botrytis cinerea, Aspergillus niger, Aspergillus flavus*, and *Fusarium graminarum*) [17, 18] as well as phytopathogenic bacteria (such as *Xanthomonas campestris* and *Erwinia carotovora*) [17].

Data reporting LAB isolation from soils and plants remain scarce. However, environmental and wild LAB strains are theoretically good competitors for different growth factors and production of antagonistic compounds but often undervalued. Olive tree is one of the most important crops in Tunisia, from North to the South but also accompanied all the Mediterranean civilizations. It is recognized for its beneficial effects on human health, even by olive oil or by different derived products [19]. Moreover, truffles are ectomycorrhizal consumable tuber, which are typical of semiarid land and are known by their important economical income for local population and their good taste [20, 21]. The specificity of rhizospheric samples for bacterial isolation is their direct contact with both plant and soil, but especially because the associated bacteria have coevolved with plant pathogenic bacteria and fungi. This study aimed to isolate LAB from olive tree and desert-truffle rhizospheric soils and to evaluate their biotechnological properties. 

## 2. Materials and Methods

### 2.1. Samples and Microbial Strains Origin

LAB strains were obtained from rhizospheric samples (49) which were collected from 8 sites located in the following regions in Tunisia: Jendouba, Ben Arous, Tunis, Kairouan, Gafsa, kebeli, Gabes, and Mednine. Samples were collected in sterilized bags, kept in cool box (<10°C) containing ice packs during the transport to laboratory, and processed within 7 days. Different other microbial species were used in antimicrobial testing. *Pseudomonas savastanoi *knW2, *Pantoea agglomerans *kn45, and* Stenotrophomonas maltophilia* KnT2 were previously isolated from olive knots [22]. *Listeria monocytogenes* L15 and *Botrytis cinerea* were obtained from the Laboratory of Microorganisms and Active Biomolecules (LMBA), Faculty of Sciences of Tunis. *Penicillium expansum* and *Aspergillus niger* from Laboratory of Microbial Ecology and Biotechnology, University of Paul Cézanne, France and *Verticillium dahliae* from the National Institute of Agronomic Research of Tunis (INRAT). Reference strains from the American Type Culture Collection (ATCC) were also used including* Enterococcus faecium* ATCC 19434, *Enterococcus faecalis* ATCC 29212,* Staphylococcus aureus *ATCC 25923, and* S. aureus* ATCC 6538.

### 2.2. Lactic Acid Bacteria Isolation Procedure

The LAB from rhizospheres were isolated by the accumulation method as described by Chen et al. [23], with some modifications. Samples of 1 g were aseptically transferred into tubes of 15 mL containing 5 mL of MRS broth (Biolife) and incubated in anaerobic candle jars at 30°C for 3 days. After incubation, samples were serially diluted in 0.75% NaCl solution. Fractions of 0.1 mL of the dilutions ranging between 10^−5^ and 10^−8^ were plated in duplicate on the surface of MRS agar (Biolife) [24] supplemented with 0.0025% of bromocresol green (MP Biomedicals) and 0.01% cycloheximide (MP Biomedicals) to inhibit fungal growth. The plates were incubated in the same conditions. The different colonies of acid-producing bacteria, determined by a yellow zone in the media around each colony, were picked and purified on MRS agar. Gram-positive and catalase-negative isolates were selected and maintained in broth with 25% glycerol at −80°C for further identification. The isolates were also tested for gas production from D-glucose (Bio Basic) (using inverted Durham tubes in MRS broth), growth at different temperatures (10 and 45°C), different pH (4.0 and 9.6), and different concentration of NaCl (3, 6.5, 8, and 9%) in MRS broth. 

### 2.3. DNA Extraction and PCR Amplification of 16S-23S rDNA Internal Transcribed Spacer and the 16S rDNA Gene

DNA was extracted by using a CTAB/NaCl method described by Wilson [25] and modified by using 1 mg/mL lysozyme (BIOMATIK) for cell wall digestion. DNA electrophoresis was performed on a 0.8% agarose gel and visualized under UV light according to the standard procedure of [26]. The PCR amplifications were performed using a thermal cycler (Thermal Cycler; Bio-Rad).

The bacterial collection was dereplicated by fingerprinting analysis of the rRNA 16S-23S intergenic transcribed spacer (ITS) region, using universal primers, s-d-bact-1494-a-20 and s-d-bact-0035-a-15 [27]. The ITS-PCR amplification consisted of 1X PCR reaction buffer, 1.5 mM MgCl_2_, 0.2 mM of dNTPs mixture, 0.5 *μ*M of each primer, 1 U Taq polymerase (Fermentas), and 150 ng of total DNA, using the following program: 94°C for 3 min, followed by 35 cycles of 94°C for 45°C, 55°C for 1 min and 72°C for 2 min, and a final extension step at 72°C for 7 min. Strains exhibiting the same band patterns were grouped in the same ITS-haplotype. One or two representative strains from each group have been selected for subsequent identification using 16S rRNA genes sequencing. The 16S rRNA amplification was performed using the describe primers s-d-bact-0008-a-S-20 and s-d-bact-1495-a-A20 [27] and the thermal profile as mentioned previously. The ITS-PCR amplification and 16S products were migrated, respectively, on 2 and 1.5% agarose gels in 0.5 × Tris-borate-EDTA buffer (reagents from Fluka-Biochemika) and stained with ethidium bromide (Sigma-Aldrich). 

### 2.4.  16S rDNA Gene Sequencing and Phylogenetic Analysis

The 16S rDNA PCR amplicons were purified with Exonuclease-I and Shrimp Alkaline Phosphatase (Exo-Sap, Fermentas, Life Sciences) following the manufacturer's standard protocol. Sequence analyses of the purified DNAs were performed using a Big Dye Terminator cycle sequencing kit V3.1 (Applied Biosystems) and an Applied Biosystems 3130XL Capillary DNA Sequencer machine. Sequence similarities were found by BLAST analysis [28] using the GenBank DNA databases (http://www.ncbi.nih.gov) and the Ribosomal Database Project (RDP). Phylogenetic analysis of the 16S rRNA gene sequences were conducted with Molecular Evolutionary Genetics Analysis (MEGA) software, version 5 [29]. Trees were constructed by using neighbor-joining method [30]. 

### 2.5. Nucleotide Sequence Accession Numbers

The sequences of the 16S rDNA gene of rhizospheric LAB isolates samples have been submitted to the GenBank databases under accession numbers KC568531 to KC568560.

### 2.6. Antibacterial Activity of LAB against Pathogen, Food-Borne, and Phytopathogenic Bacteria

The antibacterial activity test was performed using the agar-well-diffusion method described by Tagg and McGiven [31]. Five bacterial strains were used as indicators to evaluate the antibacterial activity of LAB, involving *S. aureus *ATCC6538, *L. monocytogenes *L15, *St. maltophilia*, *Ps. savastanoi,* and *Pa. agglomerans*. The cell-free supernatants (CFS) of LAB culture (48 h) in MRS broth were tested. All indicator strains were grown in BHI broth at 37°C. Trypticase soy agar plates were overlaid with 5 mL of soft agar (0.75%) containing 50 *μ*L of freshly grown culture. The wells were made in agar and filled with 100 *μ*L of the tested strain CFS. After incubation at 37°C for 18 h, the diameter of the inhibition zones was measured. The spectrum of inhibitory effect of LAB was than evaluated on indicator bacteria: *E. faecium* ATCC19434, *E. faecalis* ATCC 29212, *S. aureus *ATCC 6538, and* S. aureus *ATCC 25923. All antibacterial tests were performed in triplicate. 

### 2.7. Antifungal Activity of LAB

The LAB isolates were tested against four phytopathogenic fungi of *Aspergillus niger*, *Penicillium expansum*, *Botrytis cinerea,* and *Verticillium dahliae* using the method described by Whipps [32] with some modifications. A dual culture of the tested pathogen fungi and the presumed antagonist LAB was established in MRS agar without sodium acetate (MRS-SA). A mycelium plug of 5 mm was taken from the peripheral edge of old cultures (5 days) on PDA plates of fungal pathogens and each plug was placed at the centre of three replicate MRS-SA plates. Bacteria were inoculated at 2 cm line from the edge of plates and allowed to grow at 30°C for 48 h. Untreated control plates were plated with pathogen plugs only. Particularly for *V. dahliae,* the fungus was placed on MRS-SA five days in advance, due to its relatively slower mycelium growth. All plates were incubated on adequate growth temperature of the fungi, and the percentage of growth inhibition was calculated by using the formula of Whipps [32]: [(R1 − R2)/R1]∗100, where R1 is the radial distance (mm) grown by phytopathogenic fungi in direction of the antagonist and R2 is the radial distance grown by phytopathogenic fungi. 

### 2.8. Exopolysaccharide Production and Proteolytic Activity

The exopolysaccharide production (EPS) was evaluated by streaking fresh culture of LAB isolates on MRS agar supplemented with 2% (w/v) of sucrose (Sigma, Life science). After incubation at 30°C under anaerobic condition for 72 h, development of a mucoid colony on agar medium or long filaments (when the colony is extended with an inoculation loop) indicated the production of exopolysaccharides [33]. As well, LAB were assessed for proteolytic activity by agar-well-diffusion test in MRS containing 4% of skimmed milk (Scharlau). The diameter of the proteolysis zone was determined after incubation under anaerobic conditions at 30°C for 72 h and examined for clear zone around the wells.

### 2.9. Antibiotic Susceptibility

The antibiotic susceptibility was tested by disk diffusion method on BHIA as recommended by the standard criteria (CLSI, 2010). The antibiotics used (Bio-Rad Laboratoires, Hercules, CA, USA) for susceptibility of enterococci were ampicillin (AM; 10 *μ*g), ciprofloxacin (CIP; 5 *μ*g), chloroamphenicol (C; 30 *μ*g), erythromycin (E; 15 *μ*g), gentamycin (GM; 120 *μ*g), streptomycin (S; 300 *μ*g), tetracycline (TE; 30 *μ*g), teicoplanin (TEI; 30 *μ*g), and vancomycin (VAN; 30 *μ*g). Antibiotic discs were placed on solid media and incubated at 37°C for 24 h. Based on the inhibition zone size, the results were interpreted as resistant (R), intermediate resistant (IR), or susceptible to the antimicrobial agents (S).

## 3. Results and Discussion

### 3.1. Lactic Acid Bacteria Isolation

LAB isolates were initially selected based on their ability to produce lactic acid by the presence of yellow halo surrounding the colonies on MRS-bromocresol green plates. Only Gram-positive strains exhibiting the absence of catalase and oxidase activity were kept on MRS agar for further identification. In total, 119 LAB strains were isolated from rhizospheric samples of desert truffles (4) and olive trees (49) from diverse geographic regions in Tunisia (Table 1). LAB are usually isolated from fermented products of animal and vegetable origin. However, low rate of “somnicells” of LAB [34] are naturally found in different environments which are close to these biota, such as floor of henhouse, rhizosphere of fruit trees, and around horse barn [23, 35]. Although LAB isolation from soil and water remains scarce [23, 36], their presence in rhizospheric samples seems to be more supported by the abundance of root exudates [37].

### 3.2. Ribotyping and Identification of Isolates by 16S rRNA Gene Sequence Analysis

**Figure 1:**
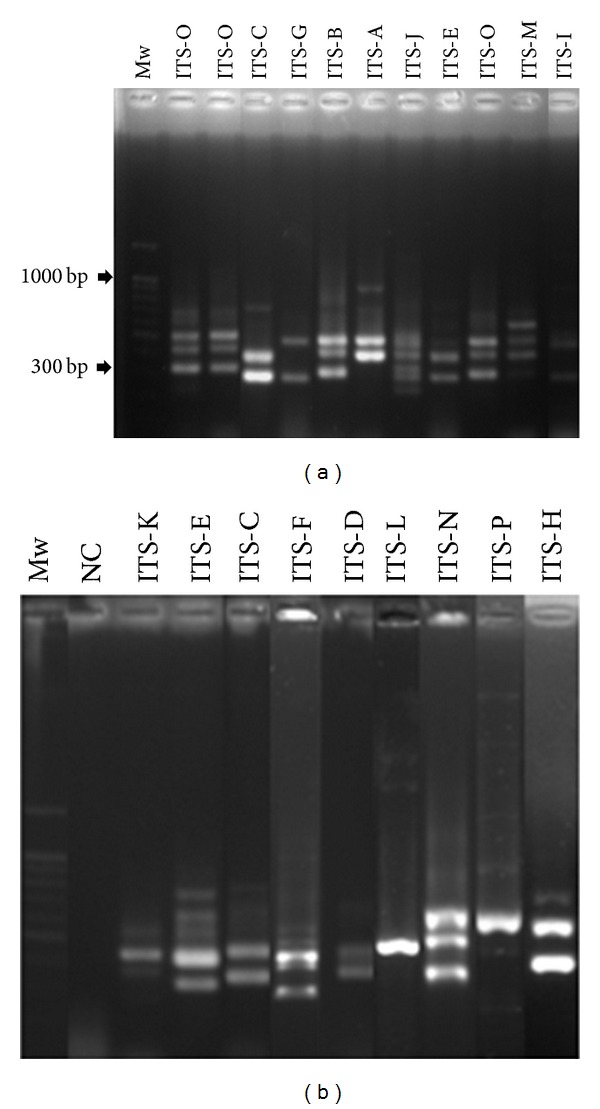
Different ITS-haplotypes (A–P) of representative rhizospheric lactic acid bacteria. Mw, Molecular weight (100 bp); NC, negative control.

**Figure 2:**
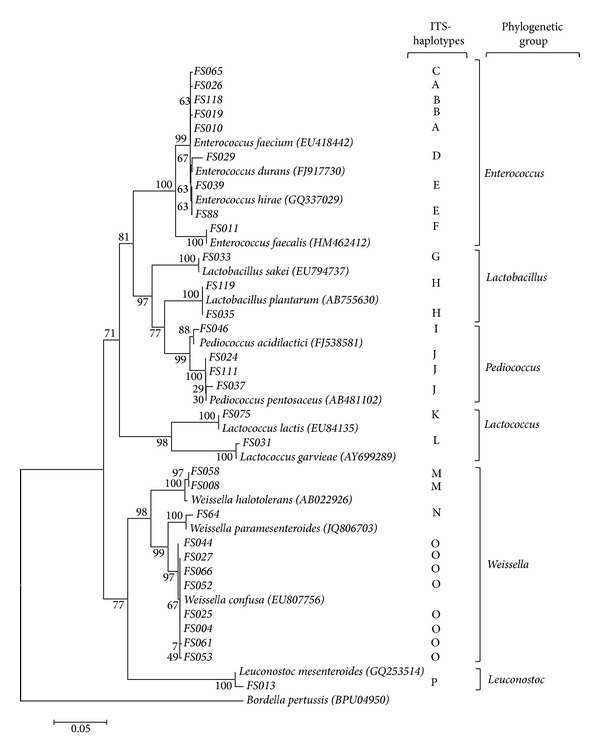
Phylogenetic tree showing the relative position of lactic acid bacteria isolates based on 16S rDNA partial sequences, using the neighbor-joining method. *Bordetella pertussis* was used as an out group. Bootstrap values for a total of 1000 replicates are shown at the nodes of the tree, using MEGA-5. The scale bar corresponds to 0.05 units of the number of base substitutions per site.

Length polymorphism analysis of amplified 16S-23S internal transcribed spacer (ITS) was used to select representative strains of the different taxonomic units issued from the LAB collection. In fact, different studies have previously reported the usefulness of ITS dereplication for inter- and intradifferentiation at the genus/species level [38, 39] due to the high variability of these internal spacers. Based on this method, 16 different ITS-haplotypes designated from A to L were distinguished. ITS-PCR patterns showed 1 to 4 reproducible bands ranging from 275 to about 600 bp (Figure 1). The representative isolates of each ITS-type (30 isolates) were identified at species level by 16S rDNA gene sequence analysis and compared to the known sequences in GenBank. Phylogenetic relationship between LAB was constructed based on the 16S rDNA sequences from evolutionary distances by the neighbor-joining method (Figure 2). Phylogenetic analysis revealed the differentiation of 5 clusters (I–V) and 11 subclusters that include members of the genera *Enterococcus, Lactobacillus, Pediococcus, Lactococcus*, *Weissella,* and *Leuconostoc*. The cluster I formed by the strains of *Enterococcus* genus was divided into 2 groups. The first group included three species of *E. faecium*, corresponding to the ITS-types (A, B, and C), *E. durans* (ITS-type D), and *E. hirae* (ITS-type E). The second group was only represented by the strain (FS11) of *E. faecalis *being the most closely related species in 100% of bootstrap analyses. The cluster II grouped strains of the genera *Lactobacillus *and *Pediococcus *and presented four subclusters (3 to 6) of *Lb. sakei*, *Lb. plantarum*, *Pc. acidilactici, *and *Pc. pentosaceus *based on the ITS-types G, H, I, and J, respectively. The cluster III formed by the strains of the genus *Lactococcus *was divided into two subclusters (7 and 8) of *L. lactis *(ITS-type K) and *L. garvieae *(ITS-type L). Furthermore, strains of the genus *Weissella *were grouped in the cluster IV, including three subclusters (9 to 11) of *W. halotolerans *(ITS-types M), *W. paramesenteroides *(ITS-types N), and *W. confusa *(ITS-types O). The cluster V was represented by one strain of *Ln. mesenteroides *(ITS-type P). The used typing method showed that almost all the identified species were represented by one ITS-type, except for *E. faecium*, which showed an intraspecies heterogeneity with three major ITS-types (A, B, and C). In fact, *E. faecium* genome is extremely diverse [40] showing a high plasticity, due to the abundance of mobile genetic elements [41]. This result is in accordance with data published by Naïmi et al. [42], Park et al. [43], and Brtkova et al. [44], which reports the ITS region variability for* E. faecium* species. Together with *W. confusa*, this species was found to be the most isolated bacterium from rhizospheric samples (Table 1). Although enterococci are normal inhabitants of the human and animal gastrointestinal tract [44, 45], they are widely distributed in nature due to their high adaptation to various environmental conditions such as food, plants, water, and soil [46]. Moreover, the isolation of bacteria belonging to the genus *Weissella* was already reported either from soil [19] or plants [17]. With regard to others studies on LAB recovery from soil [19, 35, 47, 48] a higher number of species diversity is recorded in olive tree and truffle rhizospheric samples such as *Lb. sakei, Pc. acidilactici,* and* W. halotolerans*. These species are naturally found on several raw fermented food products of plant and animal origin [49–51]; moreover, *Pc. acidilactici* is emerging as a potential probiotic in animal and human [52].

### 3.3. Physiological and Technological Properties of LAB

The physiological and biochemical characteristics including salt tolerance, growth at different temperatures, and gas production from glucose of all the strains are presented in Table 2. The majority of isolated strains were coccoid and coccoid-rods and only 5.9% showed rod shape. The majority of isolates were homofermentative, and only 13 (10.9%) were heterofermentative. From the total isolates (*n* = 79) 66.4% and (*n* = 10) 8.4% of bacterial isolates grew well in low activity water, 8 and 9% NaCl, respectively. The LAB strains with high tolerance to 9% NaCl belonged to *W. halotolerans* (FS58), *W. confusa* (FS66, FS44, FS53, FS54, and FS63), *L. lactis* (LFS20), *Lb. sakei* (FS62), and *E. faecium *(FS77 and FS103). All LAB isolates grew well in pH 4.0 and 10°C. A total of 28 (23%) and 14 (11.5%) of isolates were not able to grow in pH 9.6 and 45°C, respectively. Besides, LAB were screened for proteolytic activity and EPS production on MRS medium containing sucrose and skim milk, respectively. Results showed that all LAB isolates exhibited proteolytic activities in the cell-free supernatants as revealed by a clear halo surrounding the wells. However, the proteolytic activity varied among the strains according to the halo diameters. In fact, the more proteolytic strains (58.8%) exhibited a diameter greater than 15 mm. The most proteolytic activity (19 mm of diameter) was recorded for the strain* Pc. acidilactici* FS46. The exopolysaccharide production was detected in 42.8% of the isolates (Table 2). The good EPS-LAB producers belonged mainly to the species *W. confusa* (12 strains), *W. paramesenteroides* (FS60 and FS45), and *Ln. mesenteroides* FS13. The recorded physiochemical properties of the isolated rhizospheric LAB, for instance proteolytic activity, tolerance to high NaCl concentration, and the EPS production could explain their survival in such oligotrophic environments. In particular, EPSs are typically correlated with bacterial resistance and protection against different stress conditions such as desiccation, salt stress, and UV radiations [53, 54]. But it also generally implicated in their adherence to biological surface and sodium toxicity reduction [55]. 

### 3.4. Antibiotic Susceptibility Testing

The antibiotic susceptibility of the isolated was mainly checked for enterococcal species, since they are the predominant isolates in the collection (Table 3) and could present a risk for antibiotic resistance gene dissemination. Rhizospheric enterococci showed low percentage of resistance to chloramphenicol (3.75%), erythromycin (3.75%), streptomycin (7.5%), and tetracycline (8.75%). Nevertheless, all the strains were susceptible to teicoplanin, ampicillin, and gentamicin. Furthermore, some strains (3.7%) exhibited intermediate resistance to vancomycin and a high frequency of resistance to ciprofloxacin (36.2%). In summary, the rhizospheric enterococci showed a low frequency of resistance to the Gram-positive target antibiotics compared to food, clinical, and animal isolates [56, 57]. This result indicates the safety of these bacteria for a potential technological application. 

### 3.5. *In Vitro* Screening of the Antagonistic Activity of LAB against Human, Plant, and Food-Borne Pathogenic Bacteria

**Figure 3:**
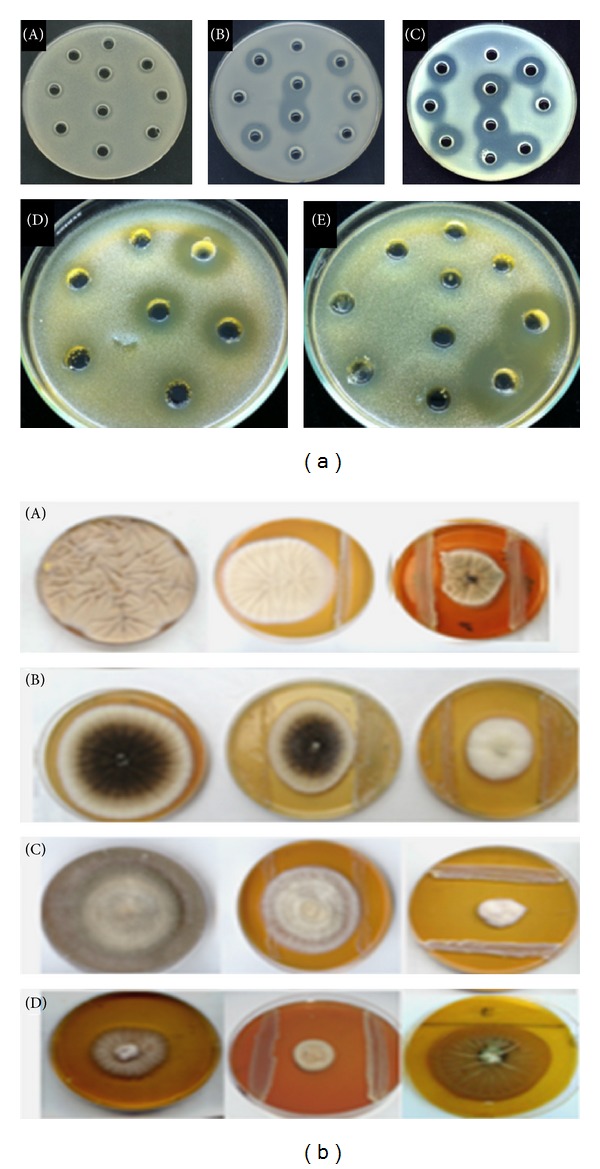
Antimicrobial activity of some rhizospheric LAB against pathogenic bacteria (a) *Pa. agglomerans* (A), *St. maltophilia* (B), *Ps. savastanoi *(C), *L. monocytogenes* (D), and *S. aureus* (E) by-agar well-diffusion method [31] and phytopathogen fungi (b) *P. expansium *(A), *A. niger* (B), *B. cinerea *(C), and *V. dahliae *(D) by dual culture [32].

**Figure 4:**
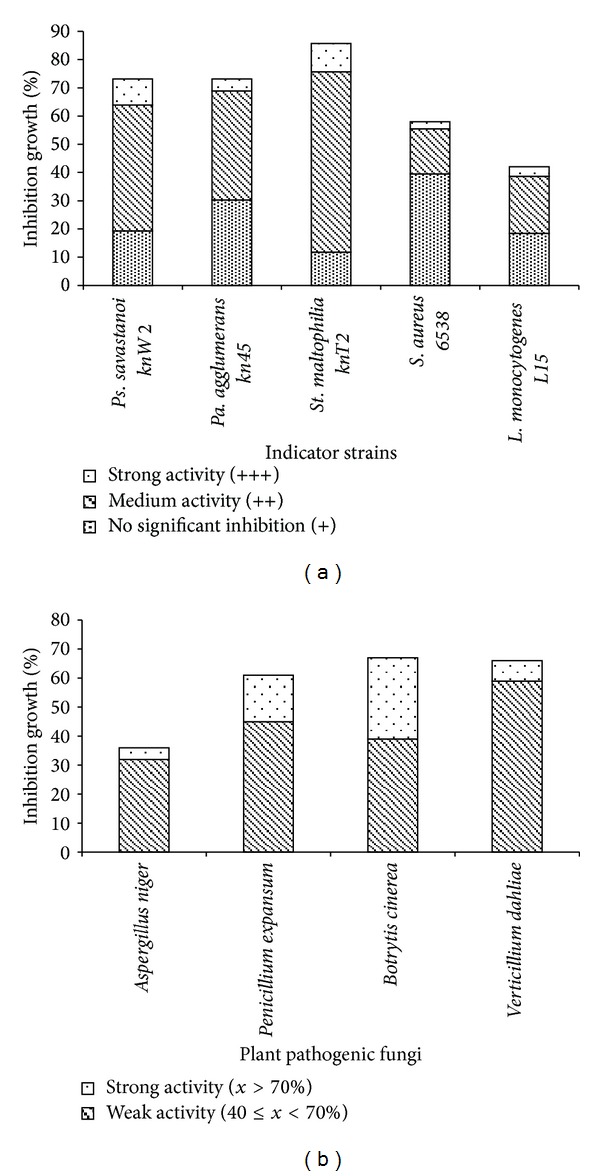
Histograms showing percentage of LAB having *in vitro* inhibitory effect on pathogenic and spoilage bacterial species (a) and plant pathogenic fungi (b). The experiments were repeated at least three times.

LAB isolates were screened for antibacterial activity against human and plant pathogens, including *St. maltophilia, Pa. agglomerans,* and *Ps. savastanoi* and food-borne bacteria of *S. aureus* and *L. monocytogenes *(Figure 3(a)). According to their inhibitory effects on pathogens, LAB were differentiated into three classes: strong inhibitor (with growth inhibition diameter (*d*) ≥ 19 mm), medium (14 ≤ *d* < 19 mm), and with no significant inhibitory effect for a diameter less than 14 mm (Figure 4(a)). The results showed that 64 strains (53.8%) have significant inhibition against *St. maltophilia*, among them 12 strains (10%) with strong inhibitory activity. This activity was recorded for the species of *Lb. plantarum, Lb. sakei, Lc. garvieae, Ln. mesenteroides, *and *Pc. pentosaceus* and mostly for the genera *Enterococcus* and *Weissella*. Five isolates (4%) including three *E. faecium *(FS70, FS01, and FS03) and two *Pc. pentosaceus *(FS73 and FS24) showed strong inhibitory activity against *Pa. agglomerans*. Eleven isolates belonging to species *W. confusa*, *Lc. Lactis, Lb. plantarum*, *Ln. mesenteroides*, *E. durans, *and *E. faecium* showed also strong inhibitory activity against *Ps. savastanoi *(diameter varied between 19 to 28 mm). The recorded high level of inhibition highlights the biotechnological potential of rhizospheric-LAB to control phytopathogens, particularly for *Pa. agglomerans* and *St. maltophilia*, which are also increasingly identified as important cause of nosocomial human infections [58, 59] and among the emergent multidrug resistant Gram-negative bacteria [59]. With regard to the food-borne pathogen, efficient inhibition was recorded for *W. confusa* FS054 strain (28 mm) against *S. aureus*, for* E. faecium* FS106 against *L. monocytogenes *(20 mm) and for the strain* W. confusa* FS036 against both *S. aureus* (20 mm), and *L. monocytogenes *(24 mm). It is of interest to note that the genera of *Enterococcus *and *Weissella *may become potential biopreservation agents of food-poisoning and plant-borne species.

This antibacterial activity exhibited by the majority of strains especially toward Gram-negative bacteria may be due to the organic acid effect or to other compounds active in acidic conditions. For this purpose, the inhibitory effect was checked after supernatant neutralization. By this way, only three strains of *E. faecium *FS071, *Ln. mesenteroides* FS013, and *W. halotolerans *FS008 retained the inhibition ability against the tested pathogens (Table 4), leading to suggest the presence of bacteriocin-like substances. This result was also supported by the broad spectrum known for the majority of the identified enterocins [60, 61]. Further studies should be conducted to elucidate the nature of the antibacterial metabolites produced by selected LAB, especially by *W. halotolerans* FS008 strain. Moreover, different studies proposed that enterococci may have a prospectively useful role in some dairy products, due to their proteolytic and lipolytic activities, and may then contribute to the development of the organoleptic properties of fermented foods, and also due to the production of enterocins with anti-*Listeria* activity [62, 63]. 

### 3.6. Antifungal Activity of LAB

LAB isolates were screened for antifungal activity against soil-borne fungi of *B. cinerea *and *V. dahliae *and postharvest contaminants of *A. niger* and *P. expansum* on MRS-SA agar medium (Figure 3(b)). According to their degree of mycelium growth reduction, active LAB were classified into two main groups: low (reduction of mycelium growth between 40 and 70%) and high antifungal activity (>70% of inhibition) (Figure 4(b)). The results showed that the maximum growth inhibition rate (28% of the strains) was registered for *Botrytis cinerea *(Figure 4(b)). The group of LAB strains with strong inhibition activity (75.3 to 92.6% inhibition) belonged to the species of *W. paramesenteroides, W. confusa, E. durans, E. faecium *and *E. hirae*. Besides, the most inhibitor strains toward the fungus *A. niger *were *E. durans *FS29 and four *E. faecium,* (FS50, FS06, FS48, and FS87) exhibiting an inhibition rate between 76.7 and 90%. Furthermore, we noted that 16% of the strains belonging to the species *E. faecium*, *E. durans*, *W. halotolerans,* and *Lb. plantarum* showed a strong inhibition rate (75.3 to 87.8%) toward *P. expansum*.

It is worth mentioning that strains of *Enterococcus* genus confirmed their antimicrobial efficacy by strong inhibition of most of the tested postharvest fungi (*P. expansum, B. cinerea,* and* A. niger*) and highlight the potential use of rhizospheric-LAB as biocontrol agents to prevent postharvest deterioration caused by these fungi. In fact, most of these fungi produce allergenic spores and mycotoxins which are responsible of the spoilage and poisoning of foods leading to serious potential health hazards [64]. It is also the case of* Lb. plantarum *FS119 which was isolated from desert truffle rhizosphere, and that could be considered as a potential candidate inhibitor of *P. expansum,* the agent of blue mold in apples. In addition strains of the genus *Weissella *have showed an efficient inhibition toward either pathogenic bacteria, or the different tested fungi. This result is in accordance with Valerio et al. [65] and Lee et al. [66] reporting the emergence/selection of these bacteria as biocontrol agent and potential probiotic. Regarding the vascular wilt fungi *V. dahlia*, Enterococcal strains were also shown to be the most efficient inhibitor strains. The highest activity was observed for *E. faecium* FS82 with 75% of mycelium reduction (Table 5). This result constitute a first report on the strong inhibition of this soil-born-fungus, which is responsible of Verticillium wilt, a serious worldwide disease that affects many crops including fruits, vegetables, and oilseed rape and leads to dramatically yield losses [67]. Biological compounds investigation to control this pathogen is of great significance [68], as it persists in the soil and resists different chemical treatments. The present study showed that selected environmental LAB could offer an excellent source for active metabolite to control different pathogenic bacteria and fungi. As it was reported by many authors [5, 13, 69], different substances, such as organic acids, hydrogen peroxide, cyclic dipeptides, and phenolic and proteinaceus compounds could be responsible for the detected antifungal activity. Indeed, identification of the issued rhizospheric-LAB metabolites is needed for a more target application. 

## 4. Conclusion

In this study, we reported for the first time the isolation and characterization of LAB from rhizosphere samples of olive trees and desert truffles. The results showed a high rate of antimicrobial activity among the isolates, indicating that rhizosphere may be a common source for the selection of LAB with important technological potential, which are useful for the biocontrol of food-, plant-, and soil-borne pathogenic bacteria and fungi. Further investigations to elucidate the nature of inhibiting compounds should be considered.

## Figures and Tables

**Table 1:** Origin and identification of rhizospheric LAB isolates.

Geographical position	Sampling point	Source/number of soil samples	Number of isolates	Strains	Closest 16S rDNA sequence	Strains (access number)	% of sequence similarity
Northeast Tunisia	Ben Arous	R. of olive tree/03	4	FS01	*Enterococcus hirae *		
				FS02	*Lactococcus lactis *		
				FS03	*Enterococcus faecalis *		
		SSR of olive tree 01		F S04	*Weissella confusa *	FS04 ( KC568542)	99
	Tunis	R. of olive tree/03	11	FS07	*Lactococcus lactis *		
				FS08	*Weissella halotolerans *	FS08 (KC568554)	99
				FS11	*Enterococcus faecalis *	FS11 (KC568559)	99
				FS13	*Leuconostoc mesenteroides *	FS13 (KC568533)	97
				FS14	*Enterococcus durans *		
				FS09, FS10, FS12, FS15	*Enterococcus faecium Enterococcus faecium *	FS10 (KC568539)	99
		SSR of olive tree/01		FS05, FS06			

Northwest Tunisia	Jendouba	R. of olive tree/21	50	FS16, FS17, FS19, FS21, FS25, FS26, FS30, FS40, FS48, FS50, FS51, FS55, FS56, FS57, FS65	*Enterococcus faecium *	FS25 (KC568541), FS19 (KC568549) FS26 (KC568553), FS65 (KC568552)	99
				FS18	*Enterococcus faecalis *		
				FS20	*Lactococcus lactis *		
				FS24, FS37, FS38, FS41	*Pediococcus pentosaceus *	FS24 (KC568551), FS37 (KC568550)	99
				FS46	*Pediococcus acidilactici *	FS46 (KC568555)	98
				FS29, FS32, FS42, FS49	*Enterococuus durans *	FS29 (KC568547)	98
				FS22, FS31	*Lactococcus garviae *	FS31 (KC568548)	99
				FS33, FS34, FS62	*Lactobacillus sakei *	FS33 (KC568535)	100
				FS35, FS59	*Lactobacillus plantarum *	FS35 (KC568557)	99
				FS39, FS43,	*Enterococcus hirae *	FS39 (KC568531)	99
				FS45, FS60, FS64	*Weissella paramesenteroides *	FS64 (KC568556)	99
				FS58	*Weissella halotolerans *	FS58 (KC568532)	99
				FS36, FS44, FS52, FS53, FS54, FS61, FS63	*Weissella confusa *	FS61 (KC568543), FS53 (KC568544), FS52 (KC568545),	99
		SSR of olive tree/04		FS23, FS27, FS28,	*Weissella confusa *	FS 27 (KC568537)	99
				FS47	*Enterococcus faecium *		

The middle Tunisia	Kairouan	R. of olive tree/03	9	FS69	*Lactobacillus sakei *		
				FS66	*Weissella confusa *	FS66 (KC568540)	99
				FS73	*Pediococcus pentosaceus *		
				FS67, FS68, FS70, FS71, FS72, FS74	*Enterococcus faecium *		

South Tunisia	Gafsa	R. of olive tree/02	3	FS76	*Weissella confusa *		
				FS75	*Lactococcus lactis *	FS75 (KC568534)	99
		SSR/01		FS77	*Weissella confusa *		
	Kebili	R. of olive tree/06	23	FS81, FS86, FS100	*Enterococcus hirae *		
				FS78, FS79, FS80, FS82, FS83, FS84, FS85, FS87, FS88	*Enterococcus faecium *	FS88 (KC568538)	99
				FS89, FS90, FS91, FS92, FS93, FS94, FS95, FS98, FS99			
		SSR/01		FS96, FS97	*Enterococcus faecium *		
	Mednine	R. of olive tree/03	7	FS101, FS102, FS103, FS105, FS106, FS107	*Enterococcus faecium *		
				FS104	*Pediococcus pentosaceus *		
		R. of truffle/04	12	FS111	*Pediococcus pentosaeus *	FS111 (KC568560)	98
		(Terfezia boudieri/Pichoa)		FS119	*Lactobacillus plantarum *	FS119 (KC568558)	99
				FS110	*Enterococcus durans *		
				FS112	*Enterococccus hirae *		
				FS108, FS109, FS113, FS114, FS115, FS116, FS117, FS118	*Enterococcus faecium *	FS118 ( KC568536)	99

R: Rhizosphere samples; SSR: soil surrounding rhizosphere; the underlined strains refer to LAB isolated from the SSR.

**Table 2:** Phenotypic characteristics of representative Gram-positive rhizospheric-LAB isolates.

	*E. faecium *	*E. durans *	*E. hirae *	*E. faecalis *	*Lb. sakei *	*Lb. plantarum *	*Pc. acidilactici *	*Pc. pentosaceus *	*Lc. lactis *	*Lc. garvieae *	*W. halotolerans *	*W. paramesenteroides *	*W. confuse *	*Ln.mesenteroides *
ITS types	A, B, C	D	E	F	G	H	I	J	K	L	M	N	O	P
Number of strains	64	06	07	03	04	03	01	07	03	03	02	03	12	01
Shape	cocci	cocci	cocci	cocci	rods	rods	cocci	cocci	cocci	cocci	coccobacilli	coccobacilli	coccobacilli	cocci
Fermentation type	Homo	Homo	Homo	Homo	Homo	Homo	Homo	Homo	Homo	Homo	Hetero	Hetero	Hetero	Hetero
Catalase	−	−	−	−	−	−	−	−	−	−	−	−	−	−
Growth at pH														
4	+	+	+	+	+	+	+	+	+	+	+	+	+	+
9.6	+	+	+	+	+	+	−	−	−	−	+	+	+	+
Growth in NaCl														
3%	+	+	+	+	+	+	+	+	+	+	+	+	+	+
6.5%	+	+(03)	+(04)	+(02)	+(03)	+	+	+(06)	+(03)	+	+	+	+	+
8%	+(42)*	+(03)	+(01)	+(01)	+(03)	+	+	+(04)	+(02)	+(01)	+	+	+	+
9%	+(02)	−	−	−	+(01)	−	−	−	+(01)	−	+(01)	−	+(05)	−
Growth at temperature														
10°C	+	+	+	+	+	+	+	+	+	+	+	+	+	+
45°C	+	+	+	+	−	+	+	+	−	−	−	−	−	−
EPS production	+(28)	−	−	−	−	+	+	−	+(02)	+(02)	−	+(02)	+	+
Proteolytic activity	+	+	+	+	+	+	+	+	+	+	+	+	+	+

(*x*): number of strains; +: positive; −: negative; Homo: homofermentative; Hetero: heterofermentative.

**Table 3:** Antimicrobial susceptibility of the enterococci isolated from the rhizosphere soils.

Antibiotics		*E. faecium *	*E. faecalis *	*E. durans *	*E. hirae *	% resistance
		(*n* = 64)	(*n* = 3)	(*n* = 6)	(*n* = 7)	
Penicillins	AM	0	0	0	0	0
Aminoglycosides	GM	0	0	0	0	0
	TE	6	0	1	0	8,75
	S	6	0	0	0	7,5
Chloramphenicols	CH	3	0	0	0	3,75
Macrolides	E	2	1	0	0	3,75
Glycopeptides	VA	3	0	0	0	3,75
	TEI	0	0	0	0	0
Fluoroquinolones	CIP	28	1	0	0	36,25

AM: ampicillin, GM: gentamicine, TE: teteracyclin, S: streptomycin, E: erythromycin, C: chloramphenicol, VA: vancomycin, TEI: teicoplanin, and CIP: ciprofloxacin. *n*: total number of strains; numbers indicated resistant strains within species.

**Table 4:** Antibacterial activity spectrum of neutralized cell-free supernatant of three LAB rhizospheric isolates.

Strains		*L. monocytogenes *L15	*S. aureus* ATCC 6538	*S. aureus* ATCC 25923	*E. faecium* ATCC 19129	*E. faecalis* ATCC 29212
*Leuconostoc mesenteroides *	FS013	21 ± 1.00	14 ± 1.00	12 ± 1.00	13 ± 1.00	10 ± 0.00
*Weissella halotolerans *	FS008	17 ± 1.00	15 ± 1.00	20 ± 1.00	13.5 ± 1.00	11 ± 1.00
*Enterococcus faecium *	FS071	16 ± 1.00	15 ± 1.05	19 ± 1.73	14.5 ± 0.80	15.5 ± 1.32

Numbers indicated the diameter of the inhibition zone in mm; each value represents the mean value standard deviation (SD) from three trials; values in the same column differ significantly (*P* < 0.05).

**Table 5:** Mycelium growth inhibition of four pathogenic fungi by selected potent antifungal rizospheric-LAB isolates using confrontation assay.

Strains	Species	*A. niger *	*P. expansum *	*B. cinerea *	*V. dahlia *
**FS29**	***E. durans***	++ (76.7)*	++ 80.2*	++ (85.1)*	++ (70.3)*
**FS50**	***E. faecium***	++ (79.1)*	+	++ (82.7)*	+
**FS06**	***E. faecium***	++ (79)*	++ (75.3)*	+	+
**FS87**	***E. faecium***	++ (79.1)*	+	++ (80.2)*	+
**FS48**	***E. faecium***	++ (90)*	++ (72.8)*	+++ (82.7)*	**+ **
**FS82**	***E. faecium***	**+**	**+**	+++ (85.2)*	+++ (75)*
FS68	*E. faecium *	+	+	++ (75.3)*	+
FS05	*E. faecium *	+	++ (80.2)*	++ (72.8)*	+
**FS14**	***E. durans***	+	++ (77.7)*	++ (82.7)*	−
FS21	*E. faecium *	+	++ (77.7)*	+	++ (70.9)*
**FS101**	***E. faecium***	+	++ (75.3)*	++ (79)*	+
**FS32**	***E. durans***	+	++ (72.8)*	++ (82.7)*	+
FS107	*E. faecium *	+	−	++ (80.2)*	+
FS45	*W. paramesenteroides *	−	+	++ (81.4)*	+
FS61	*W. confusa. *	−	+	++ (85)*	−
FS19	*E. faecium *	+	+	++ (77.7)*	+
**FS94**	***E. faecium***	**+**	++ (87.6)*	++ (82.7)*	**+**
**FS119**	***Lb. plantarum***	−	++ (83.8)*	++ (70)*	**+**
FS49	*E. durans *	−	++ (75.3)*	+	+
FS58	*W. halotolerans *	+	++ (80.2)*	+	−
**FS74**	***E. faecium***	−	++ (72.8)*	++ (85.2)*	++ (74.5)*
**FS51**	***E. faecium***	+	++ (72.8)*	++ (82.7)*	++ (70.9)*
FS102	*E. faecium *	−	+	++ (81.2)*	+
**FS16**	***E. faecium***	−	−	++ (92.6)*	−
**FS99**	***E. faecium***	−	**+**	++ (91.3)*	**+**
FS42	*E. durans *	+	+	++ (85.1)*	+
FS12	*E. faecium *	+	+	++ (75.3)*	+
FS65	*E. faecium *	+	+	++ (77.7)*	+
FS15	*E. faecium *	+	+	+	++ (70.3)*
FS106	*E. faecium *	+	++ (70.3)*	+	+
FS53	*W. confusa *	−	++ (71)*	+	+
FS77	*E. faecium *	−	++ (78.4)*	+	+

*A. niger*:* Aspergillus niger, P. expansum*:* Penicillium expansum, B. cinerea*:* Botrytis cinerea*, *V. dahlia*:* Vercticillium dahliae.* (+): weak antifungal activity having an inhibition rate between 40 and 70%; (++): strong activity with an inhibition rate ≥ 70%; the strains characterized with a broad range against different fungi appear in bold. Data were obtained at least three replicates. *Means within column show statistically significant difference (*P* < 0.05) with a control (nonexposed to the bacteria).
